# Control Using Genetically Modified Insects Poses Problems for Regulators

**DOI:** 10.1371/journal.pntd.0001495

**Published:** 2012-01-31

**Authors:** Michael J. Lehane, Serap Aksoy

**Affiliations:** 1 Liverpool School of Tropical Medicine, Liverpool, United Kingdom; 2 Yale School of Public Health, New Haven, Connecticut, United States of America

Insects are the pre-eminent form of metazoan life on land, with as many as 10^18^ individuals alive at any one instant and over three-quarters of a million species described. Although it is estimated that there are as many as 14,000 species that are blood feeders [1], only three to 400 species regularly attract our attention [2]. Some of these are of immense importance to us, as vector-borne diseases still form a huge burden on both the human population (Table 1) and our domesticated animals.

**Table 1:** Vector-borne disease still forms a huge burden on humankind.

	Prevalence	At Risk	DALYs	Major Vectors
Malaria	247 M	3.3 B	39 M	Anopheline mosquitoes
Leishmaniasis	12 M	350 M	2 M	Phlebotomine sandflies
Dengue	50 M	2.5 B	616 K	Culicine mosquitoes
Lymphatic filariasis	120 M	1.3 B	5.8 M	Mosquitoes
Sleeping sickness	30 K	70 M	1.5 M	Tsetse flies
Chagas disease	10 M	25 M	667 K	Triatomine bugs

An indication of the importance of some of the vector-borne diseases afflicting man can be seen from these WHO-derived estimates (http://www.who.int/mediacentre/factsheets/en/, accessed 3 October 2011; DALYs [19]).

B, billion; K, thousand; M, million.

Much progress has been achieved in the control of some of these vector-borne diseases by targeting the vector. The following are two good examples. First, insecticide-treated mosquito nets (ITNs) have had a major impact in the control of malaria, even in some of the most difficult control settings. The evidence from large-scale assessments shows that households possessing ITNs show a 20% reduction in prevalence of *Plasmodium falciparum* infection in children under 5 and a 23% reduction in all-cause child mortality, findings that were consistent across a range of transmission settings [3]. Second, the Southern Cone Initiative has used indoor residual spraying against the domesticated triatomine vectors of Chagas disease to immense effect [4]. As a result, the overall distribution of *Triatoma infestans* in the Southern Cone region has been reduced from well over 6 million km^2^ (1990 estimates) to around 750,000 km^2^ mainly in the Chaco of northeast Argentina and Bolivia, while *Rhodnius prolixus* has been almost entirely eliminated from Central America, with all countries there now certified by the World Health Organization (WHO) and Pan American Health Organization (PAHO) as free of transmission due to this vector.

However, the emergence and spread of insecticide resistance [5] represents a challenge to these successes and to other vector control activities, the vast majority of which depend in one way or another on the use of insecticides. The need for new insecticides (or novel means to use those we already have) and for other non-insecticidal means of vector control is quite clear. A good example of our need for new means of controlling insects is seen in dengue. Without a vaccine or drugs, disease control efforts are centred on control of the vector. But, because of the life histories of the vectors involved, the methods we currently have are inadequate [6].

One non-insecticidal method of vector control, which incidentally shows much promise for dengue control, is the use of genetically modified (GM) insects. Serious discussion of whether GM insects could be used in control began as soon as transgenic insects were first produced in the 1980s [7], and a range of means by which this could be achieved have been put forward [8]. The first generation of GM insects, designed to suppress rather than replace vector populations, is now being produced. For example, the OX3604C strain of *Aedes aegypti* is designed for the control of this dengue vector [9]. Field release of GM insects is under way [10], [11], as described by Reeves and colleagues in this issue [12]. GM insects may provide great promise for new means of controlling diseases with a devastating impact on people's lives. If so, then public acceptance is likely to be a key issue in their implementation.

It seems possible that GM insect release may prove an emotive issue. While not a GM control campaign, Reeves et al. [12] point to the decade-long WHO-led sterile insect technique (SIT) programs in India that finished in a chaotic way following ill-informed but highly damaging reporting in the Indian press [13], [14]. Similarly, the problems surrounding the use of GM crops in Europe and the issues surrounding the polio vaccination campaign in northern Nigeria [15] provide evidence of the importance of carrying public opinion if potentially beneficial technologies are to be accepted. Part of the process of carrying public opinion is to ensure that adequate oversight of technologies is in place and that the public is fully informed in an appropriate manner [15]. It is clear that research on GM vector insects has reached a stage where we can expect many field releases to take place in the near future. However, despite efforts by the European Food Safety Authority (EFSA), the Convention on Biological Diversity (CBD) Ad Hoc Technical Expert Group (AHTEG), and others, it is not clear that the regulatory processes required to oversee these releases are firmly in place, a view shared by others [16]. Although it is not a GM release as neither of the organisms involved have foreign genes inserted, the recent Australian release of *Ae. aegypti* transformed with *Wolbachia* (which reduces the capacity of the mosquito to act as a vector of dengue) [17] is an interesting example of the state of regulation in this general area. The authors state “Approval for the release of *Aedes aegypti* containing *Wolbachia* was provided by the Australian Pesticides and Veterinary Medicines Authority. Considering the novelty of the proposed experiment it was not initially clear how the open release of *Wolbachia* infected mosquitoes should be regulated in Australia. Finally after considerable consultation the Australian Government chose to regulate the release under existing legislation as a Veterinary Chemical product”.

In addition to national regulation, which is likely to be most easily organized, Mumford [18] makes the point that at least some GM insect releases may require regional or international regulation because of the risk of widespread dispersal posed by the organisms. Obtaining regional or international agreements will of course add to the difficulty of developing suitable regulatory processes. If releases of GM insects are not to prove such a highly contentious issue that it interferes with testing and implementation, then the subject requires an open and full debate in the public arena and for regulatory bodies to move rapidly to have effective and transparent oversight in place.

Consequently, we are publishing the Viewpoint article and two related Expert Commentaries in this issue with the hope that they will help to open the debate more fully on the issues surrounding the regulation of GM vector releases. We have also highlighted some of the articles previously published in PLoS journals in the Genetically Modified Insect Collection (http://www.ploscollections.org/GMInsect) for our readers interested in these topics. The international community has invested heavily in the development of a strong vector biology community and also has promoted the development of GM insect technologies to control diseases devastating animals and plants alike. Our view is that healthy discussion in a public forum can help to ensure the best possible chance that the return on our investment will be high.
